# ACORN (A Clinically-Oriented Antimicrobial Resistance Surveillance Network): a pilot protocol for case based antimicrobial resistance surveillance

**DOI:** 10.12688/wellcomeopenres.15681.2

**Published:** 2020-06-01

**Authors:** Paul Turner, Elizabeth A. Ashley, Olivier J. Celhay, Anousone Douangnouvong, Raph L. Hamers, Clare L. Ling, Yoel Lubell, Thyl Miliya, Tamalee Roberts, Chansovannara Soputhy, Pham Ngoc Thach, Manivanh Vongsouvath, Naomi Waithira, Prapass Wannapinij, H. Rogier van Doorn

**Affiliations:** 1 Cambodia Oxford Medical Research Unit, Angkor Hospital for Children, Siem Reap, Cambodia; 2 Centre for Tropical Medicine and Global Health, Nuffield Department of Medicine, University of Oxford, Oxford, UK; 3 Lao-Oxford-Mahosot Hospital-Wellcome Trust Research Unit, Microbiology Laboratory, Mahosot Hospital, Vientiane, Lao People's Democratic Republic; 4 Mahidol-Oxford Tropical Medicine Research Unit, Faculty of Tropical Medicine, Mahidol University, Bangkok, Thailand; 5 Eijkman-Oxford Clinical Research Unit, Faculty of Medicine, Universitas Indonesia, Jakarta, Indonesia; 6 Shoklo Malaria Research Unit, Mahidol-Oxford Tropical Medicine Research Unit, Faculty of Tropical Medicine, Mahidol University, Mae Sot, Thailand; 7 National Hospital for Tropical Diseases, Hanoi, Vietnam; 8 Oxford University Clinical Research Unit, National Hospital for Tropical Diseases, Hanoi, Vietnam

**Keywords:** Antimicrobial Resistance, Surveillance, Clinical

## Abstract

**Background**: Antimicrobial resistance (AMR) / drug resistant infections (DRIs) are a major global health priority. Surveillance data is critical to inform infection treatment guidelines, monitor trends, and to assess interventions. However, most existing AMR / DRI surveillance systems are passive and pathogen-based with many potential biases. Addition of clinical and patient outcome data would provide considerable added value to pathogen-based surveillance.

**Methods**: The aim of the ACORN project is to develop an efficient clinically-oriented AMR surveillance system, implemented alongside routine clinical care in hospitals in low- and middle-income country settings. In an initial pilot phase, clinical and microbiology data will be collected from patients presenting with clinically suspected meningitis, pneumonia, or sepsis. Community-acquired infections will be identified by daily review of new admissions, and hospital-acquired infections will be enrolled during weekly point prevalence surveys, on surveillance wards. Clinical variables will be collected at enrolment, hospital discharge, and at day 28 post-enrolment using an electronic questionnaire on a mobile device. These data will be merged with laboratory data onsite using a flexible automated computer script. Specific target pathogens will be *Streptococcus pneumoniae, Staphylococcus aureus, Salmonella *spp *., Klebsiella pneumoniae, Escherichia coli, *and *Acinetobacter baumannii*. A bespoke browser-based app will provide sites with fully interactive data visualisation, analysis, and reporting tools.

**Discussion**: ACORN will generate data on the burden of DRI which can be used to inform local treatment guidelines / national policy and serve as indicators to measure the impact of interventions. Following development, testing and iteration of the surveillance tools during an initial six-month pilot phase, a wider rollout is planned.

## Introduction

Antimicrobial resistance (AMR) surveillance serves three main purposes: to provide local evidence for empiric treatment guidelines and clinical decision making, to characterise trends in space and time, and to serve as benchmark to measure the impact of interventions. Current AMR surveillance systems are typically passive, pathogen-focused, and based on routine antimicrobial susceptibility testing (AST) results generated by clinical microbiology laboratories, alone. These systems lack the relevant patient-level metadata and clinical syndromic denominators to appropriately inform treatment guidelines and decision making and understand the burden of drug-resistant infections (DRIs) ^1^.

Surveillance data may suffer from various biases due to lack of diagnostic stewardship (“coordinated guidance and interventions to improve appropriate use of microbiological diagnostics to guide therapeutic decisions” ^2^) and underuse of diagnostic microbiology resources, especially in low- and middle-income countries (LMICs) ^3^. Collection of samples for microbiologic testing is often not part of a standard diagnostic work-up for many clinical syndromes. This can be due to many factors, including lack of trust between clinicians and the microbiology laboratory and (national) insurance systems that do not reimburse microbiological diagnostics. Therefore, it is common for samples to be collected only in more severe cases or in case of treatment failure. This limits direct assessment and subsequent modelling of the clinically relevant impacts and burden of DRI. Microbiologists often do not receive all clinical information important for interpreting laboratory results and surveillance data, e.g. whether an infection is community- or hospital-acquired. In addition, many patients have access to over-the-counter antibiotics in the community and are often already taking these when admitted to hospital. All of these biases favour an overrepresentation of results from DRI among surveillance data ^4^. Therefore, if one were to use current surveillance results and resistance proportions to inform clinical guidelines, there is a risk of contributing to the problem of AMR rather than the solution and advocating the use of broader spectrum antibiotic regimens than would be justified if data were more representative.

In addition to the bias-related problems noted above, several key patient-level questions that are not answered adequately by passive pathogen-focussed AMR surveillance are:

- What is the impact and cost of DRI at the patient level?
- What are the patient, hospital and environmental risk factors for DRI in a particular setting?
- Which AMR-syndrome combinations are associated with the poorest outcomes in particular patient groups?

In 2014 the WHO introduced the Global Antimicrobial Resistance Surveillance System (GLASS) that provides guidance towards standardised global surveillance of AMR focusing on a number of pathogens ("bugs") and antimicrobials ("drugs") ^5^. GLASS allows submission of "sample-based" in addition to "isolate based" data. Although this approach at least offers more clinical denominator data, it is still subject to the same biases of underuse of microbiology services and lacks clinical metadata on antimicrobial use and duration of hospitalisation ^6^.

The utility of fully integrated patient and laboratory-based surveillance was highlighted in a recent Fleming Fund funded report on AMR surveillance ^7^. High-quality patient-level surveillance data from LMICs are necessary to inform models to determine the impact of AMR using large datasets and to identify opportunities for intervention ^6^. Additionally, results based on patient-level data will be critical to generate reports that resonate with policy makers, i.e. how many people die from DRI and how much does it cost? Whilst these factors argue strongly in favour of more clinically focused surveillance, especially in LMICs, successful examples of such surveillance are limited.

The purpose of ACORN and this pilot study protocol is to establish efficient and pragmatic capture of clinical data with automated linkage to corresponding diagnostic microbiology data. Clinical variable selection was informed by an AMR stakeholder workshop, held in Bangkok in May 2019. The protocol implementation package includes tools to capture site and laboratory capacity information, guidelines on diagnostic stewardship, and a web-based data visualisation and analysis platform. The surveillance protocol is summarised below and key implementation documents are included in the accompanying *Extended data* ^8– 16^.

## Protocol

### Ethics, regulatory approvals and governance

The protocol and participant information sheet has been approved by the Oxford Tropical Research Ethics Committee (OXTREC 536-19; 21 ^st^ June 2019), Cambodia National Ethics Committee for Health Research (215-NECHR; 30 ^th^ August 2019), Laos Ministry of Health – University of Health Sciences Ethics Committee (211/19; 23 ^rd^ September 2019), and National Hospital for Tropical Disease Institutional Review Board, Hanoi, Vietnam (13/HDDD-NDTU; 18 ^th^ November 2019).

Surveillance staff will ensure that the participants’ anonymity is maintained. Personal information (i.e. name and telephone number) necessary for post-discharge follow-up will not be entered into the electronic surveillance database and will be recorded only in a paper logbook (subject identification log) at the study site. This logbook will be destroyed as soon as it no longer required. Participants will be identified only by a participant ID number on other surveillance documents and electronic databases. All documents will be stored securely and only accessible by surveillance staff and authorised personnel. The surveillance will comply with the General Data Protection Regulation (GDPR), which requires that personal data must not be kept as identifiable data for longer than necessary for the purposes concerned.

### Aims and objectives

The aim of this project is to develop an efficient clinically-orientated AMR surveillance system, implemented alongside routine clinical care in hospitals in LMIC settings. The data collected will expand on WHO GLASS, to enable classification of infection syndromes and outcomes. These data will be used to estimate syndromic and pathogen outcomes along with associated costs.

The primary objective is to develop, implement and assess a hospital-based system for patient-centred surveillance of DRI. Secondary objectives are to systematically characterise DRI based on important clinical syndromes, to adequately inform treatment guidelines; to implement clinical syndrome-guided diagnostic stewardship of patients with suspected infection; and to determine the duration, cost of hospitalisation and patient outcome of DRI and non-DRI. Finally, the tertiary objective is to evaluate the feasibility and acceptability of the surveillance system and package of tools.

The first phase of the project is development and pilot implementation over six months (which started in November 2019). Pilot implementation is occurring in three locations, focusing on a narrow range of syndromes whilst the methodology is established, and will be followed by review and refinement of the surveillance procedures, tools and results.

### Surveillance design

This protocol describes collection of clinical and laboratory data of hospitalised patients with clinically suspected meningitis, pneumonia or sepsis, for surveillance purposes. Specific target pathogens will be *Streptococcus pneumoniae, Staphylococcus aureus, Salmonella spp., Klebsiella pneumoniae, Escherichia coli,* and *Acinetobacter baumannii*, i.e. the blood culture/invasive infection relevant species from WHO GLASS.

### Surveillance sites

The sites included in the pilot phase are the Angkor Hospital for Children, Siem Reap, Cambodia; Mahosot Hospital, Vientiane, Lao People’s Democratic Republic; and the National Hospital for Tropical Diseases, Hanoi, Vietnam. These University of Oxford partner/host organisations were selected on the basis that they cover primary- to tertiary-level government and non-governmental facilities, include the full spectrum of patient groups, and the investigators are familiar with their diagnostic, laboratory and data procedures.

Pre-surveillance site visits will ensure that microbiology laboratory diagnostics and existing data capture procedures meet required standards ( *Extended data* ^8^). Summary information about the site will be documented, including number of beds, clinical services, staffing levels, medical records, and investigation/management of surveillance-relevant infections ( *Extended data* ^9^).

Investigators at each site will identify appropriate personnel to be included in surveillance training and implementation activities. Surveillance staff will be asked to complete anonymous feedback questionnaires on the surveillance tools, reports, and data visualisations. Early during ACORN site implementation, clinicians working on the selected surveillance wards will be asked to participate in a knowledge, attitudes, and practices (KAP) survey covering AMR, surveillance, and infection diagnosis and management ( *Extended data* ^10^). All surveys will be opt-out and formal informed consent will not be obtained.

### Sample size

There is no formal sample size or target. This surveillance will enrol all eligible and consenting patients admitted to the surveillance wards during the surveillance period.

### Participant selection and recruitment

The surveillance population will consist of hospitalised patients of any age (children and adults) on pre-selected surveillance wards. Sites will choose 2–3 surveillance wards based on their patient populations (“Each site should identify appropriate wards for ACORN surveillance. It may be desirable to start with a small number of wards/departments (e.g. medical admissions ward and the main intensive care unit) and scale up over time. Consideration should be given to harmonisation with other surveillance activities, where possible”) ( *Extended data* ^9^). Patients with clinically suspected community- or hospital-acquired infection (CAI/HAI) are eligible to participate in the surveillance. There is no formal consent procedure, but patients will be notified of the project and will be given the option to opt out (see below).

### Surveillance procedures

***Recruitment.*** Surveillance participants will be identified, screened and those who meet the eligibility criteria will be consecutively enrolled by surveillance personnel during daily review of new hospital admissions to surveillance wards (CAI) and during scheduled weekly point-prevalence surveys on these wards (HAI). For those patients who are screened and excluded, the reason for exclusion will be recorded. A surveillance screening log will be maintained for this purpose. Surveillance staff will be trained in the protocol and relevant surveillance procedures prior to the start of the project. With the aim of improving diagnostic consistency, international standard clinical case definitions for surveillance syndromes will be used in all site-based training activities and diagnostic stewardship materials, but will not be required to be met for enrolment during the pilot phase ( *Extended data* ^11^) ^17, 18^.

***Screening and eligibility assessment.*** For CAI, a member of the surveillance team (clinician, nurse or research assistant) will review the clinical notes of each new admission on the surveillance wards Monday to Friday. The notes of patients admitted over the weekend or on public holidays shall be reviewed on the following workday. Patients in whom there is a clinical suspicion of meningitis, pneumonia, or sepsis on admission, and also those meeting formal case definitions (Appendix 1, ACORN Diagnostic Stewardship SOP/Form, *Extended data* ^11^), will be deemed eligible for inclusion in surveillance ( *Extended data* ^12^).

For HAI, patients will be identified during weekly surveys of all patients resident in a bed on the surveillance ward at 8am on the day of the survey, excluding day case patients expected to be admitted and discharged on the same day ( *Extended data* ^13^). Patients meeting the following case definition will be deemed eligible for inclusion in surveillance (based on the European Centre for Disease Prevention and Control definition ^19^):

- Day 3 of admission onwards OR (Day 1–2 AND patient discharged from acute care hospital in preceding 48 hours) OR (Day 1–2 AND patient has relevant device inserted on this admission prior to onset)
**AND**
- Clinical diagnosis of suspected pneumonia or sepsis (hospital-acquired meningitis is not expected in the pilot sites/wards), or meets formal case definition, on the day of survey OR (patient is receiving treatment AND HAI diagnosis made between Day 1 of treatment and survey day)
**AND**
- HAI syndrome was not active during the previous weekly review (i.e. HAI syndrome onset at least one day following the most recent previous survey)

Patients may be enrolled into surveillance more than once, for example for both CAI and HAI on the same admission, multiple HAI on the same admission, or multiple admissions with CAI and /or HAI.

***Informed consent.*** The research ethics committees detailed above agreed to waive the need for explicit individual informed consent as this surveillance is a minimal/negligible risk activity, consisting of implementation of accepted quality improvement tools (diagnostic stewardship) and collection and use of limited clinical data that is expected to be collected as part of standard of care. No patient samples will be collected other than for clinical diagnostic purposes. All patients admitted to participating wards will be given an information sheet with details about the surveillance ( *Extended data* ^14^). There will also be information posters visible on these wards. The information sheet and poster will inform patients regarding the purpose and procedures of the surveillance, what it will involve for the participant, and any risks involved in taking part as well as how to get more information about the surveillance. At the time of enrolment into surveillance, the patient or parent/legal acceptable representative will be approached by a surveillance team member and asked to confirm agreement for participation in surveillance. For those unable to read the information sheet, it will be read to them at this stage. It will be clearly stated that patients have the right to refuse participation at any time, for any reason, without prejudice to future care, and with no obligation to give the reason for withdrawal. It will also be stated how to withdraw from surveillance. Any patient who requests not to be included in surveillance will be recorded accordingly in the surveillance screening logbook and will be diagnosed and treated according to standard clinical care. Surveillance staff will be readily available to provide further information and answer any questions.

Formal consent will not be obtained from clinician or surveillance staff prior to completion of anonymous surveys and feedback questionnaires. It will be explained that participation in these activities is entirely voluntary and that there will be no penalty for refusal. Participants will be informed that the data will be used to help understand better the challenges to implementation of AMR surveillance and to guide further development of the protocol.

***Baseline assessments.*** On the day of enrolment, baseline clinical data will be extracted from the patient clinical records or electronic hospital information systems and by brief interview of the patient:

- Patient hospital ID code (or other locally-used unique patient identifier).
- Date of birth or age (if date of birth not known).
- Sex.
- Co-morbidity status: cancer, chronic lung disease, chronic renal failure, diabetes mellitus, malnutrition.
- Date of admission and original hospitalisation, if transferred directly from another hospital.
- Ward name and type: medical, surgical, paediatric, intensive care unit.
- Hospitalisation in the last three months before this admission: yes or no.
- Surgery in the last three months: yes or no.
- Surveillance category: CAI or HAI.
- Surveillance diagnosis: meningitis, pneumonia, or sepsis.
- Severity score on date of admission (CAI) or symptom onset (HAI): qSOFA score for adults (≥18 years) ^20^ or the “sepsis six” recognition features for children ^21^.
- Presence of medical devices: peripheral IV catheter, central IV catheter, urinary catheter, endotracheal tube.
- Microbiology: whether a blood culture was collected within 24 hours of admission (CAI) or symptom onset (HAI); whether the patient received ≥1 dose of a systemic antibiotic in the 24 hours before blood culture collected.
- Empiric antibiotic treatment: names of systemic antibiotics prescribed on date of admission (CAI) or symptom onset (HAI).

***Subsequent assessments.*** **During hospitalisation.** A surveillance clinician will review pathogen positive cases to provide further diagnostic/treatment advice to the responsible clinician. Clinical notes and electronic hospital information systems will be reviewed to capture ICD10 code for infection episode (if routinely generated by the hospital), final classification of the surveillance diagnosis (confirmed or rejected, plus likely source in sepsis cases), hospitalisation outcome and date, and the total number of days admitted to an intensive care unit during the hospitalisation ( *Extended data* ^15^).

**Day 28 assessment.** The participant or parent/legal acceptable representative will be contacted by telephone on day 28 (+/- 7 days) post enrolment to determine post-discharge health status and date of death, if appropriate. For patients enrolled more than once in a single hospital admission (e.g. a CAI and an HAI episode, or multiple HAI episodes), a single assessment will be made, 28 days (+/- 7 days) following the final enrolment date. The day 28 assessment may occur before hospital discharge ( *Extended data* ^15^).

***Investigations.*** Specimens taken in the study are those required for routine clinical care only with no extra specimens for surveillance purposes. However, treating clinicians will be reminded of good practices for investigation of patients with suspected infection ( *Extended data* ^11^). This diagnostic stewardship will include encouragement to request blood cultures on all patients meeting the surveillance case definitions, and other specimens as indicated (e.g. cerebrospinal fluid on patients with suspected meningitis), as well as appropriate radiologic investigations on selected patients (e.g. abdominal ultrasound scan on patients with sepsis and clinically-suspected liver abscess). Microbiology specimens will be processed by onsite laboratories, to identify pathogens and their antibiotic susceptibility profiles, following approved standard operating procedures (SOPs).

### Data management and analysis

**Figure 1.**
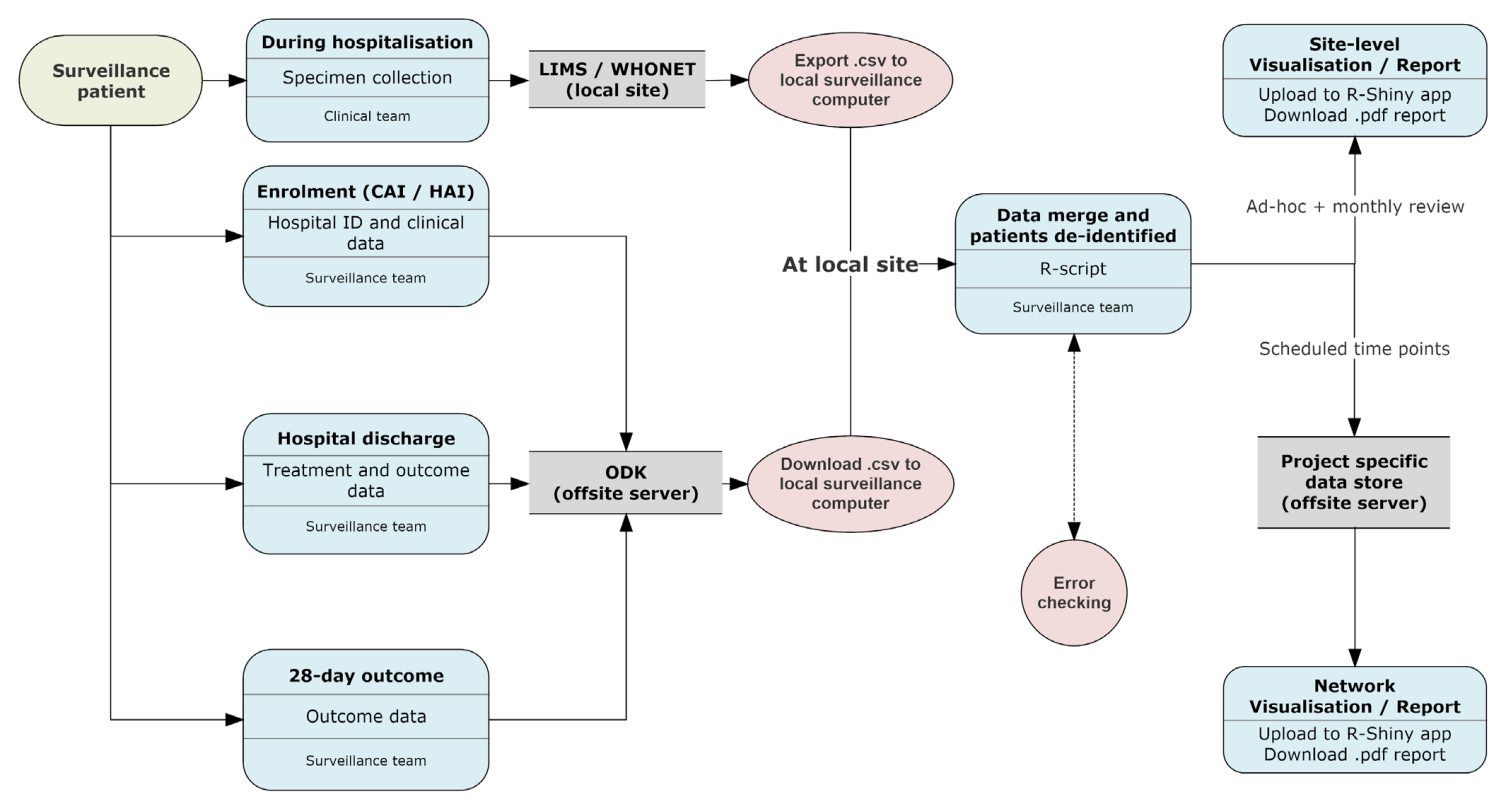
ACORN data capture and flow. CAI, community-acquired infection; HAI, hospital-acquired infection; LIMS, Laboratory Information Management System.

**Figure 2.**
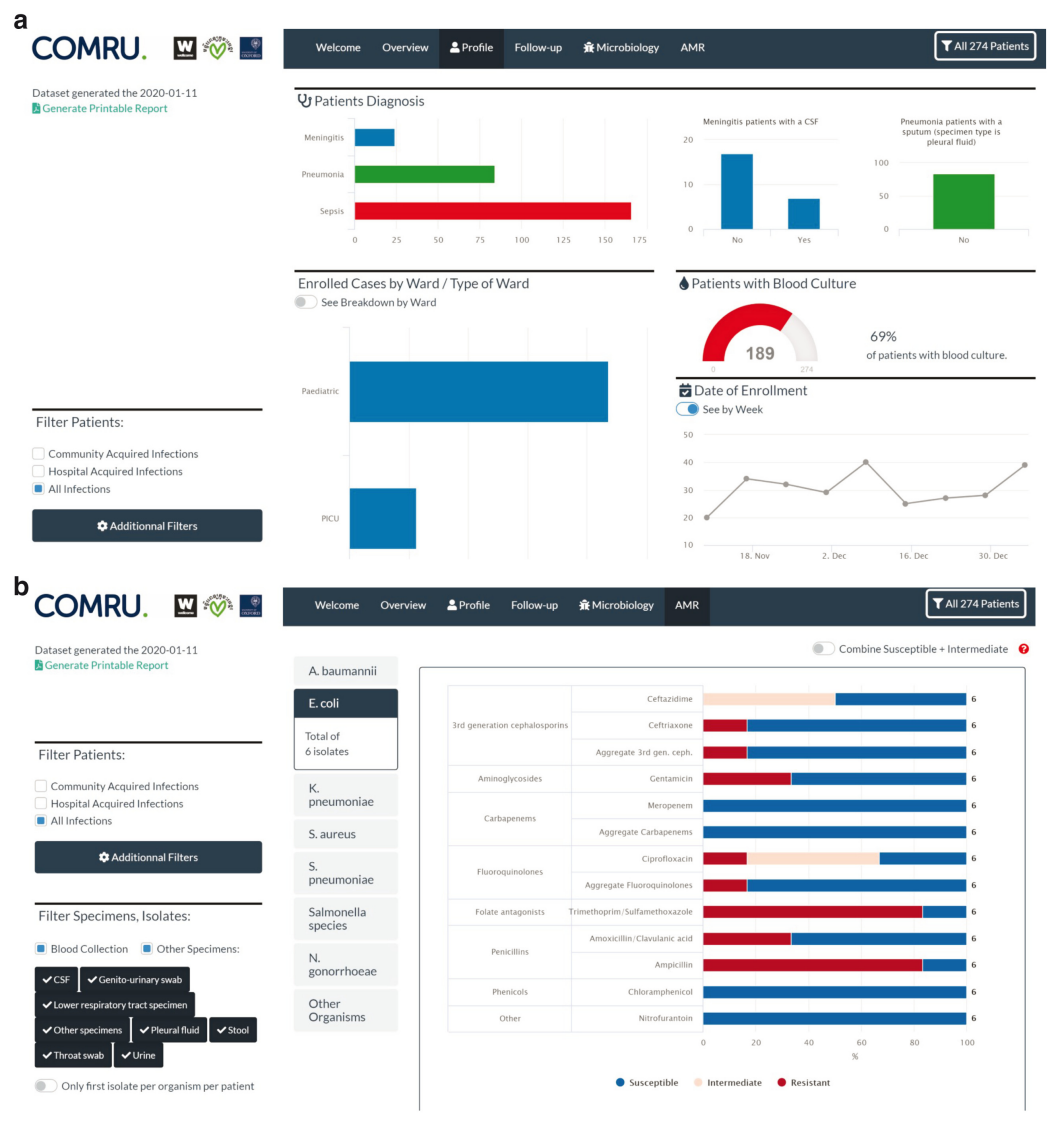
ACORN site-level data dashboard. ( **a**) Patient enrolment summary. ( **b**) Antimicrobial susceptibility summary for *Escherichia coli*.

***Data management.*** Clinical data will be captured using a short (approximately 5 minute) questionnaire on password protected Android devices via the Open Data Kit Collect (ODK) software ( Figure 1; *Extended data* ^16^). These data will be uploaded to a secure server located at the Mahidol Oxford Tropical Medicine Research Unit (MORU) in Bangkok, Thailand and downloaded periodically to password-protected computers at each site via the ODK Briefcase software. Laboratory data will be captured using the sites' existing Laboratory Information Management System (LIMS), WHONET software or using a MORU-designed ACORN LIMS (Microsoft Access) for sites with no existing LIMS. Data will be extracted from these systems into WHONET file format for further analysis. Data validation procedures will be built into the ODK forms and are included in both the ACORN LIMS and WHONET workflows. Automated script-based linkage between the two systems will be performed locally at each site using R Statistical Software ^22^. Linkages will be made using the participant hospital identification (ID) code, date of birth, and hospitalisation/specimen dates. Participant name and any other explicitly identifying detail will not be included in any analysis. Hospital sites will be identified by a unique code rather than name to reduce the possibility of linking a participant hospital ID to a specific hospital. Each time clinical and laboratory data are merged, surveillance ID will be generated for each participant and the participant hospital ID and date of birth will be deleted automatically, rendering a fully de-identified dataset for analysis and further sharing. For data visualisation and analysis, the de-identified data files will be uploaded to a secure cloud-based server and will be visualised using a bespoke R Shiny interactive dashboard ( Figure 2) ^23^. Each site will have access only to its own data, which will be available in real-time to maximise local utility.

Surveillance laboratory assessment, site survey, clinician KAP survey, and feedback questionnaire data will be captured via secure online surveys ( www.jisc.ac.uk). Only aggregate summaries of survey responses will be included in surveillance reports, presentations, and publications to ensure participant anonymity is maintained.

***AMR surveillance data analysis.*** Data will be summarised in tables and graphs on the interactive dashboard. Descriptive statistics will be used where appropriate. For syndrome-based analyses, the denominator will be the number of participants meeting the clinical case definitions. The proportions of participants in whom a blood culture and other syndrome-appropriate diagnostic specimens were collected will be calculated, along with estimation of hospital and 28-day mortality rates. For HAI prevalence, the percentage of ward patients with HAI will be determined, using the total number of patients admitted to the ward on the survey date as the denominator. For specimen-based analyses, the denominator will be the number of participants with a specific syndrome in whom a blood culture was collected. The proportions of cases in whom a pathogen was detected (specifically the selected key pathogens) or where the blood culture was considered contaminated will be calculated. For isolate-based analyses, the denominator will be the number of participants from whom a pathogen was isolated. Summaries will include the proportions of isolates resistant to key antibiotics, as defined by WHO GLASS ^5^ or categorised as multi-drug resistant, using standard definitions ^24^. Whilst the surveillance is focussed on a set of key pathogens, it is recognised that other bacterial species will be cultured from surveillance participant specimens. These species will not be included in formal analyses, but the organism and antimicrobial susceptibility data will be summarised on the interactive dashboard.

The impact of DRIs will be defined by compiling the mortality and morbidity data for patients admitted at the sites converted into disability adjusted life years (DALYs) using patient age and discharge diagnoses. The costs of their care will be estimated using data on length of stay (i.e. healthcare direct costs) and for antibiotic treatment, with hospital- and country- specific unit costs attached, respectively. These will be reported for patients with no infection, susceptible infections, and resistant infections. Modelling approaches previously described ^25^ will be applied to ascertain the incremental costs and DALYs lost that can be attributed to resistant infections as compared with susceptible or no (in the case of HAIs, assuming many of them are preventable) infections, therefore conservatively assuming that resistant infections replace, rather than add to, the burden of susceptible ones.

***Surveillance monitoring and evaluation data.*** Quantitative data will be summarised in tables and graphs. Simple descriptive statistics will be used where appropriate. Qualitative data will be reviewed to identify key themes. Clinician KAP survey data will be used to understand potential barriers to implementation of AMR surveillance at the site level as well as to contextualise the microbiologic sampling data acquired during surveillance, i.e. these data will be used to aid implementation and iteration of the surveillance activities. Feedback questionnaire data will be used to inform updates to surveillance tools.

### Dissemination

Results of the pilot will be presented at appropriate local, national and international scientific meetings and a summary manuscript will be prepared. At the conclusion of the pilot phase, an international AMR stakeholder workshop will be held to discuss the results and lessons learned during implementation of the ACORN protocol. It is anticipated that plans for the wider roll out of ACORN will be finalised during this workshop.

Software generated during the development of ACORN will be made available via GitHub at completion of the pilot phase (MS Access LIMS, ODK form templates, R-scripts to merge clinical and laboratory data, and code for the R Shiny data visualisation app).

### Study status

Enrolment commenced in Cambodia on 18 ^th^ November 2019, in Laos on 12 ^th^ December 2019, and in Vietnam on 27 ^th^ February 2020.

## Discussion

In the pilot phase, ACORN will establish patient-centred and efficient AMR surveillance at three locations within Asian LMICs. Here, preliminary data on key infection syndromes and associated pathogens will be gathered, along with identification of challenges and potential solutions to implementation of the surveillance protocol. The effectiveness of the diagnostic stewardship activities will be monitored as the proportion of cases with linked diagnostic specimens. These data will be used to iterate the protocol and tools prior to wider roll out.

Case definitions are a current area of uncertainty which will be reviewed specifically at the conclusion of the pilot phase. Clinical records are often not extensive in LMIC hospitals and the use of rigid inclusion criteria in surveillance is challenging, especially for hospital acquired infections. For this reason, very pragmatic definitions/clinical criteria have been employed in the pilot phase of ACORN, with capture of simple severity markers to enable patient stratification within a syndrome. Patients may be enrolled based on clinician diagnosis or, in the absence of clear clinician diagnosis, if they are assessed by the surveillance team as meeting the clinical case criteria for meningitis, pneumonia or sepsis. Widely used clinical definitions are used in diagnostic stewardship activities in an attempt to improve standardisation of clinician diagnosis and specimen collection. However, it is recognised that this approach may lead to limitations in inter-site data comparisons. Streamlined clinical case definitions for HAI are under development by US-CDC and WHO and these may be of use in future versions of the ACORN protocol. A hybrid solution would be to document whether a patent was included in surveillance based solely on clinical suspicion or whether a formal case definition was met, although this would require additional training and oversight. Another approach to improve specificity may be to include "prescribed antibiotics" as an inclusion criterion, i.e. specifically document whether the patient was prescribed an antibiotic. This would also add a simple antimicrobial use monitoring component to ACORN. Another area of uncertainty is around comprehensive capture of HAI cases. The use of point prevalence surveys for identification of HAI is time efficient but may result in underestimation of case numbers due to early death, discharge or recovery. However, this issue is common to all point prevalence surveys and not restricted to ACORN.

Importantly, implementation of ACORN in parallel to laboratory capacity building in LMICs will maximise the opportunities for rapid generation of actionable AMR and DRI data. It is hoped that inclusion of a simple to use data visualisation and analysis dashboard will help with local buy in and data use. In settings unfamiliar with diagnostic microbiology, without dedicated clinical support and/or surveillance activities, laboratories are likely to remain under-used and pathogen data summaries will retain current levels of ambiguity. The advantages of case-based surveillance with reporting of full AST profiles was recently highlighted ^26^. The authors commented that electronic medical records would facilitate such surveillance. These are not yet available in many LMICs and the ACORN clinical data capture tool offers an opportunity to collect such data in the absence of such systems.

In summary, ACORN will generate AMR and DRI data in LMICs that can be used to inform local treatment guidelines/national policy, in addition to inclusion in international pathogen-focused AMR surveillance systems and burden of disease studies. Generation and use of such data is a global health priority.

## Data availability

### Underlying data

No data are associated with this article.

### Extended data

Figshare: ACORN Laboratory Assessment SOP / Form. https://doi.org/10.6084/m9.figshare.11577702.v2 ^8^

Figshare: ACORN Site Preparation SOP / Form. https://doi.org/10.6084/m9.figshare.11577726.v2 ^9^

Figshare: ACORN Clinician KAP Survey / Form. https://doi.org/10.6084/m9.figshare.11577729.v2 ^10^

Figshare: ACORN Diagnostic Stewardship SOP / Form. https://doi.org/10.6084/m9.figshare.11577735.v1 ^11^

Figshare: ACORN Community Acquired Infection Enrolment SOP. https://doi.org/10.6084/m9.figshare.11577738.v1 ^12^

Figshare: ACORN Hospital Acquired Infection Point Prevalence Survey SOP. https://doi.org/10.6084/m9.figshare.11577741.v1 ^13^

Figshare. ACORN Participant Information Sheet. https://doi.org/10.6084/m9.figshare.11676453.v1 ^14^

Figshare: ACORN Patient Follow-up SOP. https://doi.org/10.6084/m9.figshare.11577747.v1 ^15^

Figshare: ACORN ODK Data Entry SOP / Form. https://doi.org/10.6084/m9.figshare.11577750.v1 ^16^

Data are available under the terms of the Creative Commons Attribution 4.0 International license (CC-BY 4.0).
