# Supplementary material for: ACORN (A Clinically-Oriented Antimicrobial Resistance Surveillance Network): a pilot protocol for case based antimicrobial resistance surveillance
Source: Wellcome Open Res. 2020 Jun 1;5:13. Originally published 2020 Jan 27. [Version 2] doi: 10.12688/wellcomeopenres.15681.2 (PMC7250055; doi:10.12688/wellcomeopenres.15681.2)
Supplement: Peer review history

# Peer review: Reviewer response for version 2

**DOI:** 10.21956/wellcomeopenres.17566.r38906

**Published:** 2020-06-02

**Authors:** Rebecca Lester

**Affiliations:** 1 Liverpool School of Tropical Medicine, Liverpool, UK

No further comments.

Is the study design appropriate for the research question?

Yes

Is the rationale for, and objectives of, the study clearly described?

Yes

Are sufficient details of the methods provided to allow replication by others?

Partly

Are the datasets clearly presented in a useable and accessible format?

Not applicable

Reviewer Expertise:

AMR/Antimicrobial Stewardship in LMICs.

I confirm that I have read this submission and believe that I have an appropriate level of expertise to confirm that it is of an acceptable scientific standard.

# Peer review: Reviewer response for version 2

**DOI:** 10.21956/wellcomeopenres.17566.r38904

**Published:** 2020-06-02

**Authors:** Maya Nadimpalli

**Affiliations:** 1 Tufts University, Medford, MA, USA

No further comments.

Is the study design appropriate for the research question?

Yes

Is the rationale for, and objectives of, the study clearly described?

Yes

Are sufficient details of the methods provided to allow replication by others?

Yes

Are the datasets clearly presented in a useable and accessible format?

Yes

Reviewer Expertise:

AMR transmission/One Health.

I confirm that I have read this submission and believe that I have an appropriate level of expertise to confirm that it is of an acceptable scientific standard.

# Peer review: Reviewer response for version 1

**DOI:** 10.21956/wellcomeopenres.17186.r38653

**Published:** 2020-05-21

**Authors:** Andrew Whitelaw

**Affiliations:** 1 Division of Medical Microbiology, Faculty of Medicine and Health Sciences, NHLS, Tygerberg Hospital, Stellenbosch University, Tygerberg, South Africa

Thanks for the opportunity to review this protocol. The protocol describes an "enhanced surveillance" system focusing on antimicrobial resistance, bringing together patient level clinical and laboratory data. The intention is to generate AMR-data that is more clinically relevant, and less subject to bias. Overall the design is appropriate and is likely to result in better quality AMR data than is currently achieved using lab-based routine data (the default position in many settings). It will also hopefully provide information that can be used to implement similar systems elsewhere

 There are a few suggestions/criticisms, but these are relatively minor, and some may not be practical.

 Page 4: The Surveillance design focuses on a set number of pathogens. It may help to clarify why these were chosen. What happens if a patient is infected with one of the other pathogens - if I understand correctly, they would still be included (or their clinical data will be included), but the organism data would not be part of the dashboard. I think this should be clarified.

 Page 4: Recruitment: the protocol states that standardised clinical case definitions will be used for training, but will not be required for enrollment during the pilot phase. I think this should be clarified. Why are case definitions not required; and is this specific to the pilot study (i.e. will case definitions be required subsequently)? The discussion makes reference to "pragmatic definitions" being used; and that the issue of case definitions will be reviewed at the end of the pilot phase. The challenge of case definitions is well understood. I am not suggesting that standardised case definitions have to or even can be used for the pilot, but I think the question of what criteria will be used for enrollment needs to be clearer. Linked to this, the definitions in SOP 6 are not clearly laid out (at least to my mind) as case definitions. They come across as clinical criteria that can be used as a guide to suggest pneumonia/sepsis/meningitis.

 Page 4/5 - HAI surveillance. This will be done as a point prevalence, weekly. The intention is to capture any patient who may have had an HAI during the previous week. What happens if a patient developed symptoms of an HAI in the preceding week, and either demised, was transferred, or recovered before the surveillance team do the weekly survey? Is there a risk of underestimating HAIs? Conversely, if a patient is included due to a clinically suspected HAI, which is subsequently shown not to be an infection, will this patient be removed from the HAI database? I suspect there is no easy way around this, and illustrates the challenge of any clinical surveillance system.

 Data management: If I understand, lab data and clinical data will be merged locally to allow for assigning the correct lab data to the correct clinical data. At that stage data will be anonymised and then further analysed. At what time point/s will this de-identification take place? Is it an ongoing process or only at the end of the pilot? Given that patients are being followed up for 28 days, and thus there are multiple sets of clinical data linked to one patient, and potentially multiple sets of lab data linked to one patient, linking the clinical and lab datasets will need to be an ongoing process, with de-identification only taking place once you are sure that no more data will be forthcoming. This is not explicitly stated - maybe it doesn't need to be.

 The study will use a MORU-designed LIMS for labs that have no electronic LIMS. Will this LIMS be accessible to clinical staff at the study sites - it may serve as a positive side effect of the study, and improve access to lab results.

 The dashboard includes provision of resistance rates for the target pathogens. The example shown has 6 *E. coli* isolates; and at a facility level the numbers of individual isolates are likely to be low (at least initially). Please be careful of displaying resistance rates for small numbers of organisms as this can be misleading. CLSI recommends at least 30 isolates before showing cumulative resistance/susceptibility rates.

 Laboratories will use either CLSI or EUCAST criteria. When merging data from different labs, this will create problems when displaying combined susceptibility data - sets of data generated using two different interpretive criteria should not be merged.

 Interpretation of susceptibility may vary depending on site of infection (penicillin for *S. pneumoniae*). How will this be managed? It would be better to record MIC data and report MICs, rather than categorical interpretation.

 Did the authors consider adding an AMS component to the study? One could include data related to whether the prescribed antibiotic for the infection was in line with guidelines; whether de-escalation occurred; whether doses were appropriate etc. (I know this adds to the workload so probably not practical - but worth asking).

 It appears that the surveillance will be done by clinical staff already employed at the hospital. Is this sustainable?

 SOP-2 - Laboratory Assessment:

 On the whole this is quite a comprehensive process. However I think there should be more detail about the AST methods used in the lab. The current form just differentiates manual and automated and asks for free text details. I would suggest tick boxes for disc diffusion, gradient diffusion MIC, broth dilution MIC. It is also complicated by the fact that many labs will use different methods for different organisms. Its not practical to capture all the variations, but identify critical ones - such as penicillin and *S. pneumoniae* which needs an MIC method.

 I would suggest a question about whether the lab is accredited to an international standard (such as ISO 15189).

 I would suggest more information about EQA - maybe review the last set of EQA results; or look for what processes are in place to monitor and resolve faults identified by the EQA process.

Is the study design appropriate for the research question?

Yes

Is the rationale for, and objectives of, the study clearly described?

Yes

Are sufficient details of the methods provided to allow replication by others?

Yes

Are the datasets clearly presented in a useable and accessible format?

Partly

Reviewer Expertise:

Clinical microbiology, with a focus on antimicrobial resistance, antimicrobial stewardship, and infection control.

I confirm that I have read this submission and believe that I have an appropriate level of expertise to confirm that it is of an acceptable scientific standard.

# Response

**Published:** 2020-05-26

**Authors:** Paul Turner

**Affiliations:** Centre for Tropical Medicine, University of Oxford, UK

**Responses to reviewer comments: Wellcome Open Research 5-13 V1**

We thank all four reviewers for their extremely helpful and constructive comments. These are all of considerable value as ACORN is iterated prior to wider roll out. Our responses are included as bullet points below.

**Reviewer 4**

Thanks for the opportunity to review this protocol. The protocol describes an "enhanced surveillance" system focusing on antimicrobial resistance, bringing together patient level clinical and laboratory data. The intention is to generate AMR-data that is more clinically relevant, and less subject to bias. Overall the design is appropriate and is likely to result in better quality AMR data than is currently achieved using lab-based routine data (the default position in many settings). It will also hopefully provide information that can be used to implement similar systems elsewhere

- We thank the reviewer for these positive comments.

There are a few suggestions/criticisms, but these are relatively minor, and some may not be practical.

Page 4: The Surveillance design focuses on a set number of pathogens. It may help to clarify why these were chosen. What happens if a patient is infected with one of the other pathogens - if I understand correctly, they would still be included (or their clinical data will be included), but the organism data would not be part of the dashboard. I think this should be clarified.

- The organisms selected as the blood culture relevant subset of the WHO GLASS organism list. This detail has been added to the "surveillance design" section of the manuscript. These will form the basis of the reporting dashboard / downloadable data summaries. However, it is possible to summarise data regarding infections due to other organisms using the dashboard (but not for now possible to download this as a word document). It is fully anticipated that additional organisms may be added to the core surveillance list as time passes (for example *Enterococcus *spp and *Pseudomonas aeruginosa*). This has been clarified by adding "Whilst the surveillance is focussed on a set of key pathogens, it is recognised that other bacterial species will be cultured from surveillance participant specimens. These species will not be included in formal analyses, but the organism and antimicrobial susceptibility data will be summarised on the interactive dashboard."

Page 4: Recruitment: the protocol states that standardised clinical case definitions will be used for training, but will not be required for enrollment during the pilot phase. I think this should be clarified. Why are case definitions not required; and is this specific to the pilot study (i.e. will case definitions be required subsequently)? The discussion makes reference to "pragmatic definitions" being used; and that the issue of case definitions will be reviewed at the end of the pilot phase. The challenge of case definitions is well understood. I am not suggesting that standardised case definitions have to or even can be used for the pilot, but I think the question of what criteria will be used for enrollment needs to be clearer. Linked to this, the definitions in SOP 6 are not clearly laid out (at least to my mind) as case definitions. They come across as clinical criteria that can be used as a guide to suggest pneumonia/sepsis/meningitis.

- We are grateful for this comment, which highlights the most challenging aspect of this project. We agree that the case definitions are worded and formatted with clinical guidance in mind and this was our preferred route for enrolment during the pilot, i.e. that these "definitions" would be used at site training to reinforce clinical diagnosis of the surveillance syndromes and that enrollable cases would thus be "clinically suspected" (but with a greater degree of standardisation). Identification of cases where clinical diagnosis is not well recorded in the medical notes has required the surveillance clinicians to assess patient fit to the case definitions as written. From site feedback, this has been relatively straightforward for pneumonia and meningitis (where the specific criteria required to make the clinical diagnosis are specified in SOP6) but, predictably, has been more of a challenge for sepsis. The manuscript discussion has been updated to: "For this reason, very pragmatic definitions / clinical criteria have been employed in the pilot phase of ACORN, with capture of simple severity markers to enable patient stratification within a syndrome. Patients may be enrolled based on clinician diagnosis or, in the absence of clear clinician diagnosis, if they are assessed by the surveillance team as meeting the clinical case criteria for meningitis, pneumonia or sepsis. Widely used clinical definitions are used in diagnostic stewardship activities in an attempt to improve standardisation of clinician diagnosis and specimen collection."
- Given the current stage of the surveillance (i.e. nearing the end of the pilot), we have not updated the study documents in response to this comment but would very much like to affirm that the point is noted and will be dealt with. On-going discussions at planned pilot investigator and stakeholder meetings will resolve this issue prior to wider rollout.

Page 4/5 - HAI surveillance. This will be done as a point prevalence, weekly. The intention is to capture any patient who may have had an HAI during the previous week. What happens if a patient developed symptoms of an HAI in the preceding week, and either demised, was transferred, or recovered before the surveillance team do the weekly survey? Is there a risk of underestimating HAIs? Conversely, if a patient is included due to a clinically suspected HAI, which is subsequently shown not to be an infection, will this patient be removed from the HAI database? I suspect there is no easy way around this, and illustrates the challenge of any clinical surveillance system.

- We agree that HAI surveillance is extremely challenging. HAI cases may be missed because of the reasons stated (indeed we note that this has happened during the pilot). Under ideal circumstances those that remain hospitalised but have recovered from their HAI within a week ought to be included, but this will require the surveillance team to maintain diligence in case identification. A comment on this has been added to the discussion: "Another area of uncertainty is around comprehensive capture of HAI cases. The use of point prevalence surveys for identification of HAI is time efficient but may result in underestimation of case numbers due to early death, discharge or recovery. However, this issue is common to all point prevalence surveys and not restricted to ACORN".
- Those patients included in HAI surveillance but subsequently determined not to infected will be identified as syndrome "rejected" at the hospital outcome data collection point. These cases can then be removed from subsequent analyses.

Data management: If I understand, lab data and clinical data will be merged locally to allow for assigning the correct lab data to the correct clinical data. At that stage data will be anonymised and then further analysed. At what time point/s will this de-identification take place? Is it an ongoing process or only at the end of the pilot? Given that patients are being followed up for 28 days, and thus there are multiple sets of clinical data linked to one patient, and potentially multiple sets of lab data linked to one patient, linking the clinical and lab datasets will need to be an ongoing process, with de-identification only taking place once you are sure that no more data will be forthcoming. This is not explicitly stated - maybe it doesn't need to be.

- Anonymisation occurs each time clinical and laboratory data are merged which enables the follow-up data to be correctly linked together prior to upload for analysis / sharing. The relevant sentence in "data management" has been updated to: " Each time clinical and laboratory data are merged, a unique surveillance ID will be generated for each participant and the participant hospital ID and date of birth will be deleted automatically, rendering a fully de-identified dataset for analysis and further sharing."

The study will use a MORU-designed LIMS for labs that have no electronic LIMS. Will this LIMS be accessible to clinical staff at the study sites - it may serve as a positive side effect of the study, and improve access to lab results.

- Unfortunately, this is not possible at the current stage of development (although the LIMS does include generation of printed and pdf clinician reports). Further development of the LIMS as part of an associated project will address this important gap.

The dashboard includes provision of resistance rates for the target pathogens. The example shown has 6 E. coli isolates; and at a facility level the numbers of individual isolates are likely to be low (at least initially). Please be careful of displaying resistance rates for small numbers of organisms as this can be misleading. CLSI recommends at least 30 isolates before showing cumulative resistance/susceptibility rates.

- We agree very much with this comment and will add an appropriate disclaimer to the dashboard. The following wording appears already in reports downloaded from the dashboard: "Care should be taken when interpreting rates and AMR profiles where there are small numbers of cases or bacterial isolates: point estimates may be unreliable."

Laboratories will use either CLSI or EUCAST criteria. When merging data from different labs, this will create problems when displaying combined susceptibility data - sets of data generated using two different interpretive criteria should not be merged.

- Thanks for this comment – we agree it is a problem. The AST criteria used by the laboratory is stored within the merged clinical-lab data file for each site and we will take care not to combine CLSI and EUCAST data.

Interpretation of susceptibility may vary depending on site of infection (penicillin for S. pneumoniae). How will this be managed? It would be better to record MIC data and report MICs, rather than categorical interpretation.

- For now the dashboard displays categorical data interpreted using the epidemiologic (i.e. meningitis) breakpoints for penicillin and *S. pneumoniae. *Moving forwards, our intention was to include a second interpretation of the penicillin data using the non-meningitis breakpoints. However, since the final site datafile retains the raw MICs (and zone sizes) alongside categorical interpretations of these, a summary table of MIC values may be a more appropriate way to display these data.

Did the authors consider adding an AMS component to the study? One could include data related to whether the prescribed antibiotic for the infection was in line with guidelines; whether de-escalation occurred; whether doses were appropriate etc. (I know this adds to the workload so probably not practical - but worth asking).

- This is very much something we are considering for future versions of the surveillance protocol. As it stands the documentation of initial treatment will at least permit sites to determine whether empiric guidelines were adhered to.

It appears that the surveillance will be done by clinical staff already employed at the hospital. Is this sustainable?

- Discussions around site-level sustainability are on-going but it will ultimately depend on resources available at the hospital and whether management / clinicians feel that the surveillance yields data of sufficient utility to allocate staff time to it.

SOP-2 - Laboratory Assessment:

On the whole this is quite a comprehensive process. However I think there should be more detail about the AST methods used in the lab. The current form just differentiates manual and automated and asks for free text details. I would suggest tick boxes for disc diffusion, gradient diffusion MIC, broth dilution MIC. It is also complicated by the fact that many labs will use different methods for different organisms. Its not practical to capture all the variations, but identify critical ones - such as penicillin and S. pneumoniae which needs an MIC method.

- Agreed - we will add this for key bug-drug combinations into the next version of this document.

I would suggest a question about whether the lab is accredited to an international standard (such as ISO 15189).

- This is included in the online document: section 12, ALAF-2: accreditation.

I would suggest more information about EQA - maybe review the last set of EQA results; or look for what processes are in place to monitor and resolve faults identified by the EQA process.

- Agreed - we will add this into the next version of this document.

# Peer review: Reviewer response for version 1

**DOI:** 10.21956/wellcomeopenres.17186.r38141

**Published:** 2020-05-20

**Authors:** Mercy Jemima Newman

**Affiliations:** 1 Department of Medical Microbiology, University of Ghana, Accra, Ghana

**Summary of the study **

1. Shows the importance of diagnostic microbiology for the treatment of systemic infections.
2. Burden of drug resistant serious infections in specific hospitals.
3. Causes and resistance of organisms of HAI and community infections.
4. It will expand on the WHO GLASS and enable classification of infectious syndromes, outcome and associated costs.
5. Surveillance training of hospital personnel.
6. Post-discharge information will be collected e.g. health status and death of patients.
7. Case definitions will be well defined at the end of the study.

**Peer Review Report **

1. Rational for the study - this is clearly described.
2. Study design - this is appropriate for the research.
3. Sufficient detail for the research has been provided.
4. Datasets are adequate.

Is the study design appropriate for the research question?

Yes

Is the rationale for, and objectives of, the study clearly described?

Yes

Are sufficient details of the methods provided to allow replication by others?

Yes

Are the datasets clearly presented in a useable and accessible format?

Yes

Reviewer Expertise:

I am a medical microbiologist. Researched on antimicrobial resistance, healthcare associated infections and investigation of disease.

I confirm that I have read this submission and believe that I have an appropriate level of expertise to confirm that it is of an acceptable scientific standard.

# Response

**Published:** 2020-05-26

**Authors:** Paul Turner

**Affiliations:** Centre for Tropical Medicine, University of Oxford, UK

**Responses to reviewer comments: Wellcome Open Research 5-13 V1**

We thank all four reviewers for their extremely helpful and constructive comments. These are all of considerable value as ACORN is iterated prior to wider roll out. Our responses are included as bullet points below.

**Reviewer 3**

Summary of the study

1. Shows the importance of diagnostic microbiology for the treatment of systemic infections.
2. Burden of drug resistant serious infections in specific hospitals.
3. Causes and resistance of organisms of HAI and community infections.
4. It will expand on the WHO GLASS and enable classification of infectious syndromes, outcome and associated costs.
5. Surveillance training of hospital personnel.
6. Post-discharge information will be collected e.g. health status and death of patients.
7. Case definitions will be well defined at the end of the study.

Peer Review Report

1. Rational for the study - this is clearly described.
2. Study design - this is appropriate for the research.
3. Sufficient detail for the research has been provided.
4. Datasets are adequate

 

- We thank the reviewer for these positive comments.

# Peer review: Reviewer response for version 1

**DOI:** 10.21956/wellcomeopenres.17186.r38656

**Published:** 2020-05-20

**Authors:** Maya Nadimpalli

**Affiliations:** 1 Tufts University, Medford, MA, USA

This protocol describes the framework for an AMR surveillance program targeted at LMIC hospitals that will combine clinical data, including follow-up data after discharge, with microbiological characterization of the etiologic agent. Combining these data streams is standard practice in many higher-income counties but not in LMICs, and successful implementation will be enormously helpful for generating accurate estimates of the prevalence of drug-resistant infections, and the mortality rate of drug-resistant versus susceptible infections. An important component of this surveillance program is that it will build trust in and demonstrate the value of routine laboratory diagnostics.

 Overall, the protocol and supporting documents were clear and well-written. I only have minor comments:

 P 3 – Please provide a brief definition or a citation for the term “diagnostic stewardship”. Clinicians and researchers based in settings where diagnostic microbiology is routinely conducted may not be familiar with the concept.

 P 4 – You mention that surveillance sites will include “the full spectrum of patient groups” and that these sites will “choose 2-3 surveillance wards based on their patient populations.” Can you be more specific? Will site + ward selection help you address one of the key questions (typically missed by passive pathogen-focused AMR surveillance) laid out on page 3 – “Which AMR-syndrome combinations are associated with the poorest outcomes in particular patient groups?”

 P 4 – For your definition of HAI, in addition to admission criteria, you write: “Clinical diagnosis of suspected pneumonia or sepsis…, or meets formal case definition, on the day of survey”. Are you referring to formal case definitions of pneumonia and sepsis? I was confused because you later discuss in detail on p. 8 how “case definitions are a current area of uncertainty.”

 P 4 – Your definition of HAI seemed a bit circular. If I am interpreting correctly, a patient could be classified as having an HAI if they met the admission criteria AND patient is receiving treatment AND HAI diagnosis made between Day 1 of treatment and survey day. In this scenario, it seems like the classification of HAI is contingent on prior HAI diagnosis. Please clarify.

 P 5 – for patients with HAIs transferred from other hospitals, how will you note a) severity score at symptom onset, b) whether a blood culture was collected at symptom onset, c) which antibiotics were prescribed at symptom onset? Will you rely on patient recall or will you be able to access prior hospital records?

 P 5- You mention in the “Day 28 assessment” section that patients can be enrolled multiple times in a single hospital admission. Please clarify this earlier, maybe in “Screening and Eligibility Assessment”.

 P 6 – For calculating HAI prevalence within a ward, you propose using the percentage of ward patients with an HAI as the numerator and the total number of patients admitted to the ward on the survey date as the denominator. It is unclear to me if this is the appropriate denominator, as not all patients admitted to the ward that day would be eligible to experience the outcome (classification as an HAI). Specifically, patients without prior hospitalization and without a device for whom disease onset occurred <3 days ago would not be eligible.

Is the study design appropriate for the research question?

Yes

Is the rationale for, and objectives of, the study clearly described?

Yes

Are sufficient details of the methods provided to allow replication by others?

Yes

Are the datasets clearly presented in a useable and accessible format?

Yes

Reviewer Expertise:

AMR transmission/One Health.

I confirm that I have read this submission and believe that I have an appropriate level of expertise to confirm that it is of an acceptable scientific standard.

# Response

**Published:** 2020-05-26

**Authors:** Paul Turner

**Affiliations:** Centre for Tropical Medicine, University of Oxford, UK

**Responses to reviewer comments: Wellcome Open Research 5-13 V1**

We thank all four reviewers for their extremely helpful and constructive comments. These are all of considerable value as ACORN is iterated prior to wider roll out. Our responses are included as bullet points below.

**Reviewer 2**

This protocol describes the framework for an AMR surveillance program targeted at LMIC hospitals that will combine clinical data, including follow-up data after discharge, with microbiological characterization of the etiologic agent. Combining these data streams is standard practice in many higher-income counties but not in LMICs, and successful implementation will be enormously helpful for generating accurate estimates of the prevalence of drug-resistant infections, and the mortality rate of drug-resistant versus susceptible infections. An important component of this surveillance program is that it will build trust in and demonstrate the value of routine laboratory diagnostics.

- Thank you.

Overall, the protocol and supporting documents were clear and well-written. I only have minor comments:

P 3 – Please provide a brief definition or a citation for the term “diagnostic stewardship”. Clinicians and researchers based in settings where diagnostic microbiology is routinely conducted may not be familiar with the concept.

- Added a definition from, and reference to, the WHO GLASS diagnostic stewardship document.

P 4 – You mention that surveillance sites will include “the full spectrum of patient groups” and that these sites will “choose 2-3 surveillance wards based on their patient populations.” Can you be more specific? Will site + ward selection help you address one of the key questions (typically missed by passive pathogen-focused AMR surveillance) laid out on page 3 – “Which AMR-syndrome combinations are associated with the poorest outcomes in particular patient groups?”

- For the ward selection, reviewer 1 made a similar comment and the response was…Suggestions for appropriate surveillance wards are made by the central surveillance team. However, sites are encouraged to make their own decisions around ward inclusion based on case mix and practical considerations (size, staffing, patient numbers). The relevant section from ref (7) has been added into the main text: "Each site should identify appropriate wards for ACORN surveillance. It may be desirable to start with a small number of wards / departments (e.g. medical admissions ward and the main intensive care unit) and scale up over time. Consideration should be given to harmonisation with other surveillance activities, where possible".
- Re. question on page 3. Yes: if one includes patient group X (e.g. adult ICU patients) and systematically collects infection and clinical data on them, then, when enough data are available, it should be possible to identify which pathogens / AMR profiles are associated with worse outcomes in that patient group, correcting for clinical things that might make a difference. At scale, inclusion of multiple sites will permit analyses which also address differences between sites. Pathogen-focussed surveillance tells us only about the bugs. Passive pathogen-focussed surveillance potentially only tells us about some of the bugs (and critically gives us no information about the patients who were not sampled).

P 4 – For your definition of HAI, in addition to admission criteria, you write: “Clinical diagnosis of suspected pneumonia or sepsis…, or meets formal case definition, on the day of survey”. Are you referring to formal case definitions of pneumonia and sepsis? I was confused because you later discuss in detail on p. 8 how “case definitions are a current area of uncertainty.”

- This is correct. For the pilot either "clinician suspicion" (i.e. whatever is recorded in the clinical notes) or surveillance staff confirmation that the patient meets the case definition / criteria (SOP6) is required for patient enrolment. Criteria for enrolling patients into AMR surveillance is indeed an area of uncertainty, especially in settings where clinician diagnoses are inconsistent, physical examination may be cursory, clinical notes are frequently incomplete, and pathology support lacking. The uncertainty is true for both CAI and HAI, but currently accepted case definitions for HAI are almost all unfit for purpose in low-resource settings.

P 4 – Your definition of HAI seemed a bit circular. If I am interpreting correctly, a patient could be classified as having an HAI if they met the admission criteria AND patient is receiving treatment AND HAI diagnosis made between Day 1 of treatment and survey day. In this scenario, it seems like the classification of HAI is contingent on prior HAI diagnosis. Please clarify.

- It isn't. The patient can be enrolled as an HAI case if they:
  - (1) meet the timing criteria
  - AND
  - (2) meet the syndromic / treatment criteria either on the day of the survey or at some point since the last survey and the day of the current survey
  - AND
  - (3) were not enrolled during a previous survey for the current HAI episode (for those HAI that have a clinical course >1 week)
- We have not encountered any user issues with the definition as stated, so have not updated in the revised manuscript.

P 5 – for patients with HAIs transferred from other hospitals, how will you note a) severity score at symptom onset, b) whether a blood culture was collected at symptom onset, c) which antibiotics were prescribed at symptom onset? Will you rely on patient recall or will you be able to access prior hospital records?

- In many settings none of these things will be able to be obtained, so they will be missed.

P 5- You mention in the “Day 28 assessment” section that patients can be enrolled multiple times in a single hospital admission. Please clarify this earlier, maybe in “Screening and Eligibility Assessment”.

- Done: "Patients may be enrolled into ACORN more than once, for example for both CAI and HAI on the same admission, multiple HAI on the same admission, or multiple admissions with CAI and / or HAI."

P 6 – For calculating HAI prevalence within a ward, you propose using the percentage of ward patients with an HAI as the numerator and the total number of patients admitted to the ward on the survey date as the denominator. It is unclear to me if this is the appropriate denominator, as not all patients admitted to the ward that day would be eligible to experience the outcome (classification as an HAI). Specifically, patients without prior hospitalization and without a device for whom disease onset occurred <3 days ago would not be eligible.

- We agree that this is a pragmatic choice but note that it is consistent with other point prevalence survey (PPS) methodologies and one that is readily standardised over time and across sites.
  - The Global PPS ( https://www.global-pps.com/) collects a "total admitted patients" denominator and then additional denominators comprising the number of admitted patients with particular devices (present on the day of the PPS).
  - The UK PHE HAI PPS data collection forms also collect total number of patients resident on ward at time of survey ( https://assets.publishing.service.gov.uk/government/uploads/system/uploads/attachment_data/file/774082/PPS_forms_flowcharts.pdf).

# Peer review: Reviewer response for version 1

**DOI:** 10.21956/wellcomeopenres.17186.r37955

**Published:** 2020-04-17

**Authors:** Rebecca Lester

**Affiliations:** 1 Liverpool School of Tropical Medicine, Liverpool, UK

This paper describes a pilot study protocol for a multi-site AMR surveillance project, which aims to prospectively link clinical and diagnostic microbiological data and which can be implemented alongside routine clinical care. The study has gained ethical approval and the pilot phase is ongoing. 

 The type of prospective AMR surveillance described by this protocol is much needed and, as the authors identify, should help to minimise the biases associated with many AMR studies which are frequently pathogen/drug specific, or which do not link microbiology to clinical data. The site-level data dashboard is a great development which will provide user-friendly and interactive data visualisation.

 p4-5: The authors begin to address this in the discussion, but is the definition of HAI/CAI too narrow/specific? Will this approach miss patients who have attended other types of healthcare setting than acute care hospitals - for example outpatient services and health centres? Likewise, will definition of community acquired be able to distinguish community onset/hospital acquired from true community onset infections? The extended data describes possible reclassification of some CA cases based on transfer and admission dates. Although standardisation across sites may be difficult, the authors might consider making a judgement on this classification prospectively at enrolment and taking into account more nuanced clinical history. 

 p5: Likewise, It's not completely clear whether a blood culture collected within 24 hours of admission (CAI) automatically put someone in CAI category? 

 p5: What will be the purpose of qSOFA and sepsis six in the analysis? It is extremely challenging to come up with a unifying sepsis severity score for all age groups, particularly in LMICs where qSOFA is not well validated, but may be worth highlighting these limitations in the discussion if sepsis severity will play an important role in the burden models. Will any blood tests be taken into account?

 General/Methods: A lot of information on methods is contained comprehensively within the SOPs in extended data. Whilst appreciating the word count limitations, could some of these be summarised in the main paper - in particular?

- Cost data could be more comprehensively described - is it only antibiotics included in the unit costs and are these healthcare direct costs or patient indirect costs as well?
- Likewise, the qualitative aspect to this study will be hugely informative but the description of this is somewhat hidden in the main text. Could this be separated out and made clearer in the confines of the work count?
- Discussion p 8: "The effectiveness and cost-effectiveness of the diagnostic stewardship activities will be monitored as the proportion of cases with linked diagnostic specimens." How does this address cost-effectiveness? Again could this be made clearer in a costing paragraph in methods?

**Minor comments:**

- Are there any references for paragraph two of the introduction, which describes the various biases associated with existing AMR surveillance data?
- What level of QC is available to the diagnostic laboratories?
- How were surveillance wards selected? Any space to explain this in the main text?
- Why were the specific target pathogens chosen? If possible a justification would be informative.

Is the study design appropriate for the research question?

Yes

Is the rationale for, and objectives of, the study clearly described?

Yes

Are sufficient details of the methods provided to allow replication by others?

Partly

Are the datasets clearly presented in a useable and accessible format?

Not applicable

Reviewer Expertise:

AMR/Antimicrobial Stewardship in LMICs.

I confirm that I have read this submission and believe that I have an appropriate level of expertise to confirm that it is of an acceptable scientific standard.

# Response

**Published:** 2020-05-04

**Authors:** Paul Turner

**Affiliations:** Centre for Tropical Medicine, University of Oxford, UK

Many thanks for the review and comments. We will address these and upload a revised manuscript once the second peer review comes in.

To anyone out there who'd like to be a peer reviewer...please don't hesitate to get in touch with the editorial team. No prizes for doing so, but we'd be very grateful.

# Response

**Published:** 2020-05-26

**Authors:** Paul Turner

**Affiliations:** Centre for Tropical Medicine, University of Oxford, UK

**Responses to reviewer comments: Wellcome Open Research 5-13 V1**

We thank all four reviewers for their extremely helpful and constructive comments. These are all of considerable value as ACORN is iterated prior to wider roll out. Our responses are included as bullet points below.

**Reviewer 1**

This paper describes a pilot study protocol for a multi-site AMR surveillance project, which aims to prospectively link clinical and diagnostic microbiological data and which can be implemented alongside routine clinical care. The study has gained ethical approval and the pilot phase is ongoing.

The type of prospective AMR surveillance described by this protocol is much needed and, as the authors identify, should help to minimise the biases associated with many AMR studies which are frequently pathogen/drug specific, or which do not link microbiology to clinical data. The site-level data dashboard is a great development which will provide user-friendly and interactive data visualisation.

- We thank the reviewer for these positive comments.

p4-5: The authors begin to address this in the discussion, but is the definition of HAI/CAI too narrow/specific? Will this approach miss patients who have attended other types of healthcare setting than acute care hospitals - for example outpatient services and health centres? Likewise, will definition of community acquired be able to distinguish community onset/hospital acquired from true community onset infections? The extended data describes possible reclassification of some CA cases based on transfer and admission dates. Although standardisation across sites may be difficult, the authors might consider making a judgement on this classification prospectively at enrolment and taking into account more nuanced clinical history.

- We appreciate this comment. Our experience is that it is incredibly difficult for clinical surveillance to be both comprehensive and done consistently / well, especially in resource limited settings. We felt that there was a need for pragmatism in this step that would inevitably result in a compromise and the potential to miss some cases. Simplified case definitions (e.g. to all patients with a fever) may increase sensitivity but this would be at the expense of specificity, which may result in overload and compromise of the entire system. That said, the enrolment criteria are the subject of on-going discussion as we approach the end of the pilot phase.
- For the second point, it was concluded that, on balance, being able to re-classify CAI as healthcare-associated based on objective evidence recent healthcare exposure and/or direct transfer from another facility was more objective / standardised compared with asking clinicians (of differing experience) to make judgement calls on the origin of infection.

p5: Likewise, It's not completely clear whether a blood culture collected within 24 hours of admission (CAI) automatically put someone in CAI category?

- This is an important point. We request that surveillance staff record these cases as “CAI”. However, as noted above, the intention is that patients will be stratified on whether they are transferred in (or have recent healthcare exposure) or are direct admissions. In this respect ACORN will be more nuanced than the simple CAI / HAI classification of WHO GLASS which is based entirely on timing of culture.

p5: What will be the purpose of qSOFA and sepsis six in the analysis? It is extremely challenging to come up with a unifying sepsis severity score for all age groups, particularly in LMICs where qSOFA is not well validated, but may be worth highlighting these limitations in the discussion if sepsis severity will play an important role in the burden models. Will any blood tests be taken into account?

- We completely agree with the difficulties around sepsis severity scores, especially for use in children and/or in LMICs where access to supporting laboratory tests is patchy. The purpose of including qSOFA and the sepsis six signs is to give an indication of patient severity, to help contextualise the clinician diagnosis. We felt that including the requirement for blood test results would be unhelpful given the desire for ACORN to be useful to settings with limited resources. This use of the severity score is noted in the discussion and will not form a major component of burden modelling – the results are intended to be more for site-level data reviews (e.g. correlation of qSOFA score against blood culture positivity). As such, we have not updated the existing statement: "For this reason, very pragmatic definitions have been employed in the pilot phase of ACORN, with capture of simple severity markers to enable patient stratification within a syndrome."

General/Methods: A lot of information on methods is contained comprehensively within the SOPs in extended data. Whilst appreciating the word count limitations, could some of these be summarised in the main paper - in particular?

Cost data could be more comprehensively described - is it only antibiotics included in the unit costs and are these healthcare direct costs or patient indirect costs as well?

- As currently stated, these calculations will include length of stay (i.e. healthcare direct costs) and antibiotic treatment: " The costs of their care will be estimated using data on length of stay and for antibiotic treatment, with hospital- and country- specific unit costs attached, respectively.". This has been further clarified by inclusion of "(i.e. healthcare direct costs)".

Likewise, the qualitative aspect to this study will be hugely informative but the description of this is somewhat hidden in the main text. Could this be separated out and made clearer in the confines of the work count?

- This component is intentionally relatively small in the pilot phase and the results are largely intended to inform updates to the surveillance. With this in mind, we prefer not to expand the current description.

Discussion p 8: "The effectiveness and cost-effectiveness of the diagnostic stewardship activities will be monitored as the proportion of cases with linked diagnostic specimens." How does this address cost-effectiveness? Again could this be made clearer in a costing paragraph in methods?

- We agree, this sentence is flawed. The words "cost-effectiveness" have been removed.

Minor comments:

Are there any references for paragraph two of the introduction, which describes the various biases associated with existing AMR surveillance data?

- Most of the biases are covered in existing reference (2). We have added the following reference, which includes a brief summary of the issues and points to further relevant references: Yam ELY, Hsu LY, Yap EP-H, Yeo TW, Lee V, Schlundt J, Lwin MO, Limmathurotsakul D, Jit M, Dedon P, Turner P, Wilder-Smith A. Antimicrobial Resistance in the Asia Pacific region: a meeting report. Antimicrobial Resistance & Infection Control. 2019;8(1):202.

What level of QC is available to the diagnostic laboratories?

- The laboratory assessment tool includes a review of quality control / quality assurance procedures in place. ACORN does not explicitly provide laboratory QC/QA but makes recommendations based on the laboratory assessment. The intention is that sites will not be included in ACORN if their laboratories are not able to meet the required quality standards. Initial site rejection could be reviewed on documentation of quality / technical improvements.

How were surveillance wards selected? Any space to explain this in the main text?

- Suggestions for appropriate surveillance wards are made by the central surveillance team. However sites are encouraged to make their own decisions around ward inclusion based on case mix and practical considerations (size, staffing, patient numbers). The relevant section from ref (7) has been added into the main text: "Each site should identify appropriate wards for ACORN surveillance. It may be desirable to start with a small number of wards / departments (e.g. medical admissions ward and the main intensive care unit) and scale up over time. Consideration should be given to harmonisation with other surveillance activities, where possible".

Why were the specific target pathogens chosen? If possible a justification would be informative.

- Target pathogens are the invasive infection / blood stream relevant organisms from the WHO GLASS surveillance scheme. This detail has been added to the "surveillance design" section of the manuscript.
